# Efficacy, chemical composition, and pharmacological effects of herbal drugs derived from *Fritillaria cirrhosa* D. Don and *Fritillaria thunbergii* Miq.

**DOI:** 10.3389/fphar.2022.985935

**Published:** 2022-11-30

**Authors:** Fan Wu, Mei Tian, Yuefeng Sun, Changhao Wu, Xue Liu

**Affiliations:** ^1^ College of Traditional Chinese Medicine, Shandong University of Traditional Chinese Medicine, Jinan, China; ^2^ Department of Respiration, Affiliated Hospital of Shandong University of Traditional Chinese Medicine, Jinan, China

**Keywords:** *Fritillaria cirrhosa*, *Fritillaria thunbergii* Miq., pharmacological action, efficacy, chemical composition

## Abstract

*Fritillaria cirrhosa* D. Don and *F. thunbergii* Miq. belong to the genus *Fritillaria* within the Liliaceae family. They are used in traditional Chinese medicines that are often administered in clinical settings as they have notable effects on cough, bronchitis, pneumonia, lung injury, cancer, and other diseases. In this review, we focus on the history, origin, similarities, and differences in efficacy, chemical composition, and pharmacological outcomes of the drugs obtained from *F. cirrhosa* (FRC) and *F. thunbergii* (FRT). We list various valuable pharmacological effects of FRC and FRT, including antitussive, expectorant, anti-inflammatory, antioxidant, and anticancer effects. Thus, this review offers a basis for the medical application of and further research into the pharmacological impacts of these two drugs. We believe that new drugs derived from the phytoconstituents of *F. cirrhosa* and *F. thunbergii* that have specific therapeutic properties can be developed in the future.

## 1 Introduction

Fritillaries are plants that belong to the genus *Fritillaria*, within the lily family (Liliaceae). The drugs obtained from these plants are called beimu (hereafter referred to as FR). The two most used species of fritillaries for medicinal purposes are *Fritillaria cirrhosa* D. Don, also known as Sichuan Fritillaria, and *F. thunbergii* Miq., also known as Bulb of Thunberg Fritillary (Nile et al., 2021). The drugs obtained from these two species are called FRC and FRT, respectively. As a commonly used traditional Chinese medicine (TCM), FR has a long history of use. There is great global demand for wild *F. cirrhosa*, which is traded in considerable quantities in China, Hong Kong, Taiwan, Canada, Malaysia, Singapore, Korea, and Europe. The demand is particularly high in China, where it exceeds the supply, leading to a price increase of 500 USD per kilogram for *F. cirrhosa* between 2002 and 2017 (Cunningham et al., 2018). The present study is the first to review the chemical composition and pharmacological effects of both FRC and FRT. It highlights the similarities between FRC and FRT concerning their pharmacological effects, providing a basis for further research into their medicinal application.

### 1.1 Historical use of fritillary in medicine

FR was first recorded in *Shen Nong’s Materia Medica* (25–200 AD), where it was listed as a middle-grade herbal medicine, based on toxicity and medicinal effect. The author, Tao Hongjing said, “The shape is like a collection of shellfish, hence the name *Fritillaria*.” According to the *Treatise on Febrile Diseases and Miscellaneous Diseases* written by Zhang Zhongjing in 200–210 AD, referred to as the ancestor of the prescription book, *Fritillaria* species have been utilized to reduce phlegm. In these initial works, FRC and FRT were not clearly distinguished; they were both first differentiated in the late Ming dynasty. “Chuan Fritillaria” was first mentioned in *Materia Medica in Southern Yunnan* (1463 AD). In the *Collected Statements on the Herbal Foundation* (1624 AD), *Fritillaria* species were further classified and compared, with the final proposition that *F. cirrhosa* and *F. tubei* (later called *F. thunbergii* Miq.) are different (Qing et al., 2005). *Fritillaria thunbergii* was first mentioned in the *Description of Materia Medica* published in 1691 AD. Thereafter, *Benjing Fengyuan* (1695 AD) and *Benjing Congxin* (1757 AD) described the clear distinction between FRC and FRT. *A Supplement to Compendium of Materia Medica* (1765 AD) explains the basic differences in therapeutic effects between the two. More than 20 medical records of FRC and FRT use are described in the *Clinical Guide Medical Records* published in 1746 AD (Li et al., 2020). Knowledge about the medicinal value of FR also spread to other East Asian countries and was documented in the *Medical Heart Formula* (Japan, 982 AD) and *Oriental Medicine Treasures* (Korea, 1611 AD) (Wang et al., 2021). Currently, medicines containing FR are sold commercially in Japan and Korea for a variety of uses. Furthermore, FR is also widely used as an herbal remedy in other Asian cultures, such as in the Indian Ayurvedic system and Arabian or Unani medicine in Central and Western Asia. However, the *Fritillaria* species used in these systems are different from the ones used in China. The historical use of FR as a medicine is further described in Table 1.

**TABLE 1:** Historical use of *Fritillaria* as a medicine.

Dynasty	Representational work	Significance
Qin and Han dynasties (221 BC–220 AD)	*Shen Nong’s Materia Medica*	Earliest record that describes the theory of pharmacy
	*Treatise on Febrile Diseases and Miscellaneous Diseases*	Clinical use of *Fritillaria*, no distinction between FRC and FRT
Wei and Jin dynasties (220–450 AD)	*Mingyi Bielu*	Still uses fritillary as the mainstream appellation
Five years in the Tengen of Japan (982 AD)	*Medical Heart Formula*	Earliest documented reference to FR in Japan
Ming dynasty (1368–1644 AD)	*Southern Yunnan Materia Medica*	First appearance of FRC in literature
	*Collected Statements on the Herbal Foundation*	Classification and comparison of efficacy
Korea (1611 AD)	*Description of Materia Medica*	First appearance of FRT in literature
	*Oriental Medicine Treasures*	Earliest record of FR in Korea
Qing dynasty (1636–1912 AD)	*A Supplement to Compendium of Materia Medica*	Distinguishing FRC from FRT

Abbreviations: FRC, drug from *Fritillaria cirrhosa*; FRT, drug from *Fritillaria thunbergia*.

### 1.2 Origins and anatomical characteristics of *Fritillaria cirrhosa* and *Fritillaria thunbergii*

**FIGURE 1:**
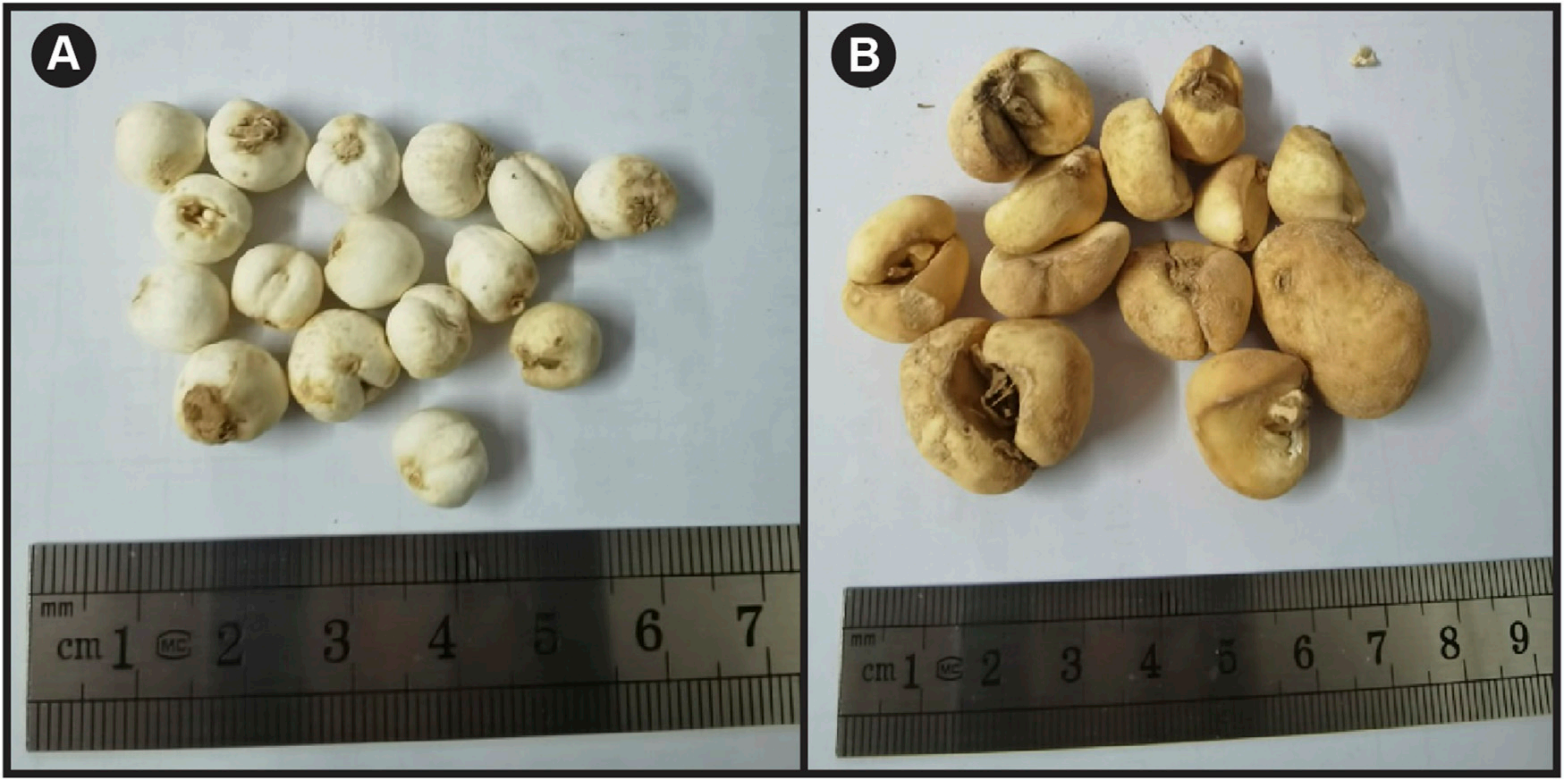
Photographs of the dried bulbs of **(A)** *Fritillaria cirrhosa* D. Don and **(B)** *Fritillaria thunbergii* Miq.

To date, 165 FR species have been identified worldwide and found in the temperate zone of the Northern hemisphere (Paudel et al., 2021). FRC and FRT medicines are described as two different entities in the Chinese Pharmacopoeia (Chinese Pharmacopoeia Commission, 2015); however, they share commonalities on a biological basis. From a botanical viewpoint, both FRC and FRT are obtained from the desiccated bulbs of plants belonging to the Liliaceae family. FRC is the desiccated bulb of *F. cirrhosa*, *F. przewalskii* Maxim., *F. unibracteata* Hsiao et K.C. Hsia, or *F. delavayi* Franch., which typically grow under bushes and in woods, fields, wetlands such as streams and seashores, valleys, and rock clefts (Chen and Helen, 2000). Crude plant extracts utilized as medication are mainly obtained from the Gansu (southern), Qinghai, Ningxia, Shaanxi (Qinling), and Shanxi (south) regions in China. Regarding FRT, in Nepal, the Kingdom of Bhutan, Japan, and other countries, dried bulbs are obtained locally from *F. thunbergii* plants that grow in the shade of lower slopes or in bamboo woodlands. In China, the bulbs are obtained from plants that grow mainly in the Jiangsu (southern), Zhejiang (northern), and Hunan regions. FRC and FRT can be distinguished based on origin, morphological characteristics, and chemical composition (Chinese Pharmacopoeia Commission, 2015). FRC and FRT have different attributes (Figures 1A,B), which can be examined *via* microscopic observation, thin layer chromatography, polymerase chain reaction-restriction fragment length polymorphism, and content determination. Some researchers have also used matrix-assisted laser desorption/ionization to identify FRs (Wang et al., 2020). According to the Chinese Pharmacopoeia, the total alkaloid content of the dry product of FRC, which is calculated by quantifying sibemrine (C_27_H_43_NO_3_), should be above 0.05%. Similarly, the total peimine (C_27_H_45_NO_3_) and peiminine (C_27_H_43_NO_3_) content of the dry product of FRT should be above 0.08% (Chinese Pharmacopoeia Commission, 2015).

As shown in Figure 1A, FRC is either conical or nearly spherical, with a height of 0.3–0.8 cm and a diameter of 0.3–0.9 cm. The surface of FRC surface is off-white in color, and the external scale leaves have two petals that are incredibly divergent in size. The large petal folds tightly around the small one, and the unfolded portion of the large petal is crescent-shaped. It is commonly said that holding the flower is like holding the moon in one’s arms. One to two small-scale leaves are present within the flower, with obtuse apices that are round or slightly pointed. The bottom of the bulb is flat and slightly concave with a gray-brown bulb disk at the center; occasionally, fibrous roots remain on the bulb. FRC is hard, brittle, and slightly bitter and has a slight smell and a white cross-section that is rich in powder.

As shown in Figure 1B, the single-lobed scale leaf on the external layer of the FRT bulb is slightly crescent in shape, 1–2 cm long, and 2–3.5 cm wide. The external surface is grayish to light yellow, while the internal surface is white or light brown, with white powder. The bulb is hard, brittle, easy to break, and slightly bitter and has a minor smell and a white to yellow-white section that is rich in powder. *Fritillaria thunbergii* slices are oval or quasi-circular with different sizes. They are typically 1.5–3.5 cm long, 1–2 cm wide, and 0.2–0.4 cm thick. The outer skin is yellowish-brown or gray-brown when slightly shrunken or pale yellow when relatively smooth. The cut surface bulges slightly and is grayish-white or is flat and pinkish-white. The slices are brittle and rich in white flour.

### 1.3 Similarities and differences in the efficacy of FRC and FRT

FRC and FRT are clinical TCMs that are widely used in the treatment of cough and respiratory diseases (Nile et al., 2021). Both FRC and FRT are classified as heat-clearing and phlegm-resolving drugs. FRC tastes bitter, is slightly cold in nature, and acts on the lung and heart meridians (Hu et al., 2020). It dissipates heat and humidifies the lung, thereby loosening phlegm and relieving cough and asthma. It is generally used for treating dry cough with lung heat, dry cough with less phlegm, tuberculosis caused by yin deficiency, blood in sputum, lung carbuncles, etc. (Liu et al., 2020a). FRC holds clinical significance for diverse pulmonary illnesses, including pneumonia and acute lung injury (Liu et al., 2020a). FRT is bitter, cold in nature, and acts on the lung and heart meridians. It dissipates heat, resolves phlegm, relieves cough, and detoxifies and dispels carbuncles. It can be used to treat cough, bronchitis, hypertension, bacterial infection, and other illnesses caused by wind-heat and phlegm-heat (Cui et al., 2018). In addition, FRT may be used to treat tumoral masses, whereas FRC has little effect on them, Therefore, FRT has been incorporated in some TCM formulations for treating cancer (Hao et at., 2013). Furthermore, FRT can also be used to treat drug-resistant leukemia (Wang et al., 2013). Furthermore, FRC is sweet and moisturizing; it can moisten the lungs and relieve cough, and is specifically appropriate for treating chronic cough caused by internal injury, dry phlegm, and hot phlegm. On the other hand, FRT is strongly bitter and efficient in clearing heat-phlegm and reducing lung-qi. Moreover, its ability to dispel knots and carbuncles is stronger than that of FRC. The differences between the properties of FRC and FRT are summarized in Table 2.

**TABLE 2:** Difference between FRC and FRT.

Classification	FRC	FRT
Categorization	Heat-clearing and phlegm-resolving medicine	Heat-clearing and phlegm-resolving medicine
Taste	Sweet but slightly bitter flavor, slightly cold	Bitter, cold
Drug meridian Channel tropism	Lung and heart meridians	Lung and heart meridians
Effects	Clearing away heat, moistening the lungs, resolving phlegm, and relieving cough	Clearing away heat, resolving phlegm, relieving cough, and detoxifying
Indications	Dry cough, expectoration, asthma, pneumonia, pulmonary carbuncle, and acute lung injury caused by lung heat	Cough caused by wind-heat and phlegm-heat, bronchitis, high blood pressure, bacterial infection, nodular mass

Abbreviations: FRC, drug from *Fritillaria cirrhosa*; FRT, drug from *Fritillaria thunbergii*.

### 1.4 Differences in the chemical composition of FRC and FRT

FRC and FRT have comparable origins, similar chemical compositions, and are together known as FR (Wu et al., 2018). Researchers have used several methods including high-performance liquid chromatography, quadrupole time-of-flight mass spectrometry, and near-infrared spectroscopy to investigate the chemical composition of FR (Meng et al., 2015; Mohammat et al., 2017). Modern pharmacochemical and pharmacological research has already proven that the primary additives that contribute to the biological potency of FR consist of isosteroidal alkaloids, steroidal alkaloids, non-alkaloids, terpenoids, and steroidal saponins responsible for antitussive activity (Lin et al., 2001; Hu et al., 2018; Wang et al., 2021). Of the 140 compounds isolated from FR, most were isosteroidal alkaloids (72.7%), followed by non-alkaloids (15.8%) and steroidal alkaloids (11.5%) (Lin et al., 2001). Differences in the place of origin and growing conditions also affect the contents of medicinal plants to a certain extent. Studies have shown that light-emitting diodes affect the steroidal alkaloid contents in fritillaries. Furthermore, red- and infrared-light exposure increases fritillary alkaloid content (Chen C. C. et al., 2020). *Fritillaria cirrhosa* is found in the wild and is also grown under artificial illumination, whereas *F. thunbergii* is primarily grown under artificial lights. Owing to the high demand for FRC in the pharmaceutical market, wild *F. cirrhosa* has been collected in large amounts and is now listed as a Grade III protected species (Zhang et al., 2010). Additionally, different harvesting periods and processing methods affect the quality and quantity of active ingredients within cultivated *Fritillaria*. The best time to harvest this herb is during the wilting period, when the total alkaloid content is at its highest (0.088–0.218%). Drying after washing also ensures quality and improves productivity (Ma et al., 2021).

The primary chemical constituents of FRC are isosteroidal alkaloids, including *F. cirrhosa* ketones, verticillin ketones, verticillin, and fritillary alkaloids (Liu et al., 2020a). Clear experimental evidence has proven the antitussive and expectorant effects of FRC-derived alkaloids, crinoids (C5), and isovitamin (C6) (Wang et al., 2012). Moreover, amide alkaloids are also found in FRC (Liu et al., 2014). According to the Chinese Pharmacopoeia, sibemidine content is mainly used as the quality control standard of FRC.

**FIGURE 2:**
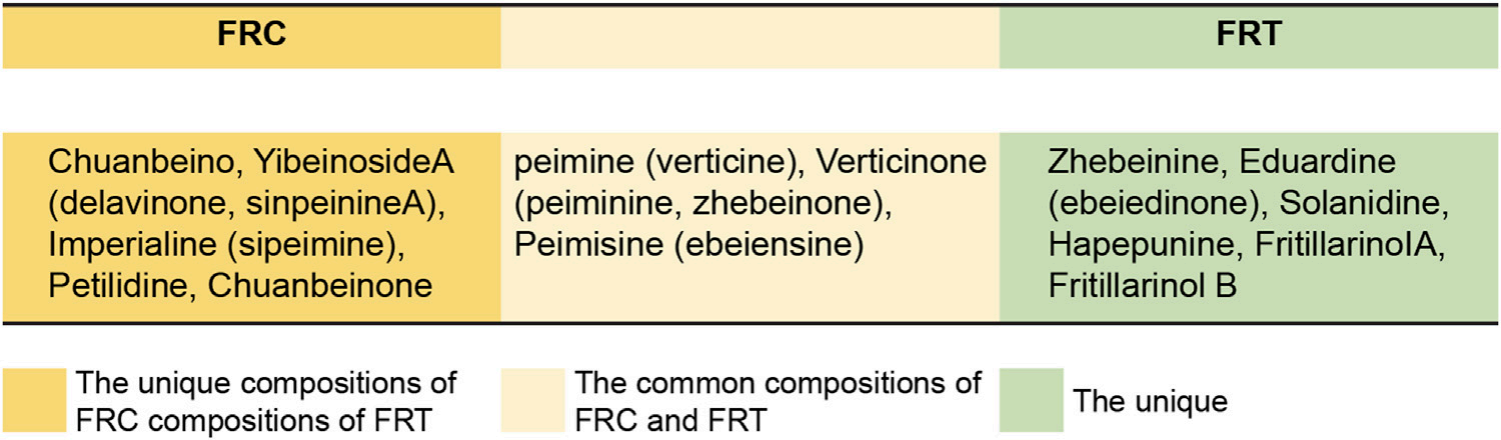
Main chemical constituents of FRC and FRT.

**FIGURE 3:**
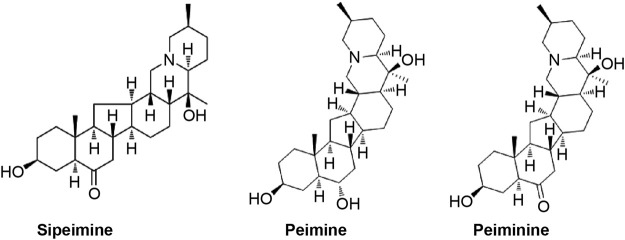
Chemical structures of sibemine, fritillium A (peimine), and fritillium B (peiminine).

FRT comprises diverse chemical elements, including flavonoids, essential oils, saponins, and alkaloids. FRT and its bulbs are mainly composed of alkaloids, essential oils, diterpenes, polysaccharides, amino acids, nucleosides, sterols, lignans, and fatty acids. According to the Chinese Pharmacopoeia, the contents of fritillium A and fritillium B are mainly used as the quality control standard of FRT. The main chemical components of FRC and FRT are illustrated in Figure 2. The chemical structures of sibemine, fritillium A, and fritillium B are illustrated in Figure 3.

## 2 Pharmacological effects of FRC and FRT

### 2.1 Pharmacological effects of FRC

Cough suppression, sputum removal, anti-asthmatic, anti-inflammatory, antioxidant, and anticancer properties are some of the pharmacological properties of FRC described in the available literature (Wang et al., 2012; Wang et al., 2016c; Chen T. et al., 2020).

#### 2.1.1 Antitussive and expectorant effects

Cough is one of the most common ailments for which people seek medical help; however, owing to the side effects of narcotic cough suppressants, the treatment options for severe cough remain unsatisfactory (Dicpinigaitis, 2015). FRC has been used to treat cough in China for over 2,000 years. Alkaloids, such as chuanbeione and progesterone isolated from *F. chinensis* can drastically prolong cough latency, reduce cough frequency in mice, and enhance the production of phenol crimson in the windpipe of mice (Wang et al., 2011). Active alkaloid-chemical sites, such as 17-βH, 22-αH, and 20-OH may also play essential roles in mediating the antitussive and expectorant properties of FRC (Wang et al., 2011). Four alkaloids in *F. cirrhosa*, namely, imperialine, imperialine N-oxide, isoverticine, and isoverticine N-oxide can dramatically inhibit cough in mice and exert an apparent antitussive effect (Wang et al., 2012). Of these alkaloids, imperialine and isortyline exhibited antitussive effects in a dose-dependent manner (Wang et al., 2012). The alkaloids present in *F. cirrhosa* can notably increase the excretion of phenol pink within the trachea of mice, which is a method for evaluating expectoration, thereby indicating that *F. cirrhosa* can relieve cough and reduce phlegm (Wang et al., 2011; Wang et al., 2012). Moreover, FRC extract increased cough latency and suppressed cough frequency in mice (Xu et al., 2019). As an anti-inflammatory compound, FRC extract also prevented the development of ear edema and enhanced the output of phenol red into the trachea of mice (Xu et al., 2019). Thus, the pharmacological outcomes of FRC in terms of relieving cough, resolving phlegm, and reversing inflammation have been demonstrated. Crude alkaloid (10^−9^–10^−5^ g/ml) and aqueous extracts (10^−7^–10^−3^ g/ml) of FRC relaxed isolated trachea and bronchi of rats subjected to carbachol-induced precontraction, in a concentration-dependent manner (Wu et al., 2018).

#### 2.1.2 Alleviation of acute lung injury and resistance to fibrosis

Acute lung injury is usually caused by direct or indirect alveolar injury, often resulting in acute respiratory distress syndrome (Hughes and Beasley, 2017). Consequently, mortality from acute lung injury is extremely high. It has been shown that inflammatory chemokines, particularly interleukin-6 (IL-6) and tumor necrosis factor-α (TNF-α), play an important role in the onset of acute lung injury (Hu et al., 2015; Rahimi et al., 2017). Wu and his collaborators showed that FRC-derived alkaloids attenuated lipopolysaccharide (LPS)-induced acute lung injury in mice with a correlated reduction in IL-6 and TNF-α levels (Wu et al., 2018). Peimine, which is found in FRC, also ameliorates acute lung injury in mice by inhibiting the expression of TNF-α, IL-1β, IL-6, and IL-8, and further reducing lipid raft formation in alveolar epithelial cells, which is beneficial in alleviating pulmonary fibrosis (Guo et al., 2013; Du et al., 2020).

#### 2.1.3 Anti-asthmatic effect

Asthma is a long-term inflammatory disease of the respiratory tract that adversely impacts the lives of children and elderly persons (Hamid et al., 2003). Experiments show that FRC can reduce nitric oxide (NO), TNF-α, IL-1, IL-6, and malondialdehyde (MDA) levels, increase the activity of superoxide dismutase (SOD), and possibly inhibit matrix metalloproteinase-2 (MMP-2), matrix metalloproteinase-9 (MMP-9), and matrix metalloproteinase-2 (MMP-2). Tissue inhibitor of metalloproteinase-1 (TIMP-1) inhibits airway remodeling in asthmatic mice Histopathological experiments have proved that imperialine contained within FRC, can substantially reduce the percentage of neutrophils in bronchoalveolar lavage fluid as well as in peripheral blood and inhibit TGF-β1 protein expression in lung tissue by correcting TIMP-1/MMP-9 imbalance. Imperialine alleviates the pathological damage and reduces bronchiolar stenosis substantially (Wang et al., 2016a). An *in vivo* study on asthmatic mice demonstrated the strong inhibitory effect of FRC on airway inflammation (Yeum et al., 2007). The aqueous extract of FRC inhibited the expression of Th2 cytokines (IL-4, IL-5, and IL-13), reduced IgE and eosinophil aggregation, and increased IFN-γ production in the bronchoalveolar fluid (Yeum et al., 2007).

#### 2.1.4 Anti-inflammatory effect

Peimine obtained from FRC exhibits considerable anti-inflammatory effects *in vivo* by inhibiting LPS-mediated inflammation. Experiments show that peimine inhibits the production of LPS-induced inflammatory cytokines by blocking the MAPKs and NF-κB signaling pathways (Yi et al., 2013). Peiminine can substantially lessen LPS-induced expression of many pro-inflammatory cytokines, including TNF-α, IL-6, cyclooxygenase-2, and inducible nitric oxide synthase, by suppressing the phosphorylation of protein kinase B (AKT) and NF-κB p65 (Chen et al., 2018). Verticinone or imperialine, also found in FRC, can dose-dependently inhibit the production of nitric oxide, expression of both nitric oxide synthase and cyclooxygenase-2, and production of pro-inflammatory cytokines such as TNF-α and IL-1β (Wu et al., 2015). The total alkaloid extract of FRC inhibited paw edema caused by carrageenan and granuloma caused by cotton pellets (Wu et al., 2015). Moreover, FRC-derived edpetiline can upregulate the expression of IL-4 and IL-10, which are cytokines that exhibit powerful anti-inflammatory effects (Zhang et al., 2021). Five alkaloids from FRC exert anti-inflammatory effects by inhibiting LPS-induced phosphorylation of MAPK signaling pathway in RAW264.7 macrophages. As this pathway comprises the anti-inflammatory mediators extracellular signal-regulated kinase (ERK1/2), p38 MAPK, and c-Jun N-terminal kinase (JNK), FRC may be an alternative drug for treating inflammation (Liu et al., 2020b). Some studies have shown that FRC can also relieve gastritis, resist fever and improve memory (Paudel et al., 2021).

#### 2.1.5 Antioxidant effect

Oxidative stress is linked to numerous diseases, including cancer, cardiovascular disease, Parkinson’s disease, Alzheimer’s disease, chronic obstructive pulmonary disease, and rheumatoid arthritis (Pizzino et al., 2017). FRC may serve as a promising therapeutic option for preventing illnesses related to oxidative stress. FRC-derived vitexinone, verticine, imperialine-3-β-D-glucoside, delavir, pemixin, and imperialine reduce the generation of reactive oxygen species, increase the production of glutathione, and promote the expression of heme oxygenase (HO-1), which induces NF-erythroid factor 2-related factor 2 (Nrf2) nuclear translocation, which in turn, is associated with Nrf2 expression upregulation (Liu et al., 2020a). Edpetiline, which is also found in FRC, was shown to reduce the level of reactive oxygen species generated during inflammation, thereby diminishing oxidative stress (Zhang et al., 2021).

#### 2.1.6 Analgesic effect

Verticinone is an alkaloid extracted from FRC, that can inhibit the torsional response triggered by acetic acid; 3 mg/kg verticinone results in a torsional response inhibition of up to 66.2%, which is higher than that induced by 200 mg/kg aspirin (Xu et al., 2011). An analgesic assessment of neuropathic pain revealed that verticinone is more stable than morphine in terms of its analgesic effect (Xu et al., 2011). These studies suggest that verticinone exerts good analgesic effects on both inflammatory and neuropathic pain, with little dependency induction. Thus, verticinone exhibits great potential as an analgesic.

#### 2.1.7 Antiviral effect

FRC extract exerts an antiviral effect by inhibiting the viral replication cycle. It is most effective against H1N1 influenza virus and does not cause toxic reactions *in vivo* and *in vitro*. In comparison with oseltamivir, FRC extract is notably less toxic and safer. The use of FRC within 24 h after viral infection resulted in higher survival rates and lower weight loss in mice (Kim et al., 2020).

#### 2.1.8 Anticancer effect

Cancer is the leading cause of death worldwide, with high morbidity and mortality (Jemal et al., 2010). Consequently, it poses a great risk to human health and economic and social development. Despite the approval of several new antitumor drugs every year, most cancer treatments remain limited (Paulson et al., 2013). Interestingly, the aqueous extract of FRC induces apoptosis in cancer cells *via* immunoregulation mediated by signal transducer and activator of transcription (STAT) 1 and 4 (Li et al., 2020). Moreover, experimental studies have confirmed that FRC-derived imperialine can reliably and safely exert anticancer effects on non-small cell lung cancer and anti-inflammatory effects on non-small cell lung cancer tumors both *in vivo* and *in vitro* (Lin et al., 2020). Furthermore, peiminine mediates cell cycle arrest by inhibiting Akt/glycogen synthase kinase 3 beta (GSk3β) and AMP-activated protein kinase (AMPK)/autophagy-activating kinase (ULK1) signaling, which leads to reduced autophagic flux, thereby slowing tumor growth down (Zhao et al., 2018). An *in vivo* study demonstrated that the alkaloids in FRC retarded the expansion of Lewis lung tumors in rats (Wang et al., 2014) by inhibiting tumor angiogenesis and stimulating apoptosis in cancer cells. The mechanism of action may involve the contribution of total alkaloids in FRC to the downregulation of caspase-3 and endothelial cell adhesion molecule-1 (Wang et al., 2014). Chuanbeinone, which is present in FRC, showed significant antitumor activity *in vivo*. This component lowered the expression of the anti-apoptotic protein B-cell lymphoma 2 (Bcl-2) and increased the expression of the pro-apoptotic Bcl-2-associated X protein (Bax) and caspase-3, thereby promoting the apoptosis of tumor cells (Wang et al., 2016b). Zheng and his collaborators reported the accumulation of various amino acids such as glutamine in a peiminine-treated colorectal cancer cell line (HCT-116), which may reflect changes in glutathione balance, suggesting that peiminine could impair redox homeostasis in cancer cells (Zheng et al., 2017). It also induced cell death in HCT-116 cells by modulating the production of metabolites such as glucose, glutamine, and oleic acid to alter the autophagic flux. A previous study revealed that FRC extracts block endometrial cancer growth by downregulating the TGF-β/SMAD signaling pathway (Bokhari and Syed, 2015). FRC-derived verticinone inhibits the proliferation of malignant oral keratin-forming cells in a dose- and time-dependent manner, mainly by arresting the G_0_ and G_1_ cell cycle and inducing apoptosis (Yun et al., 2008). Verticinone also inhibits the proliferation of human promyelocytic leukemia HL-60 cells by inducing cellular differentiation (Pae et al., 2002).

**FIGURE 4:**
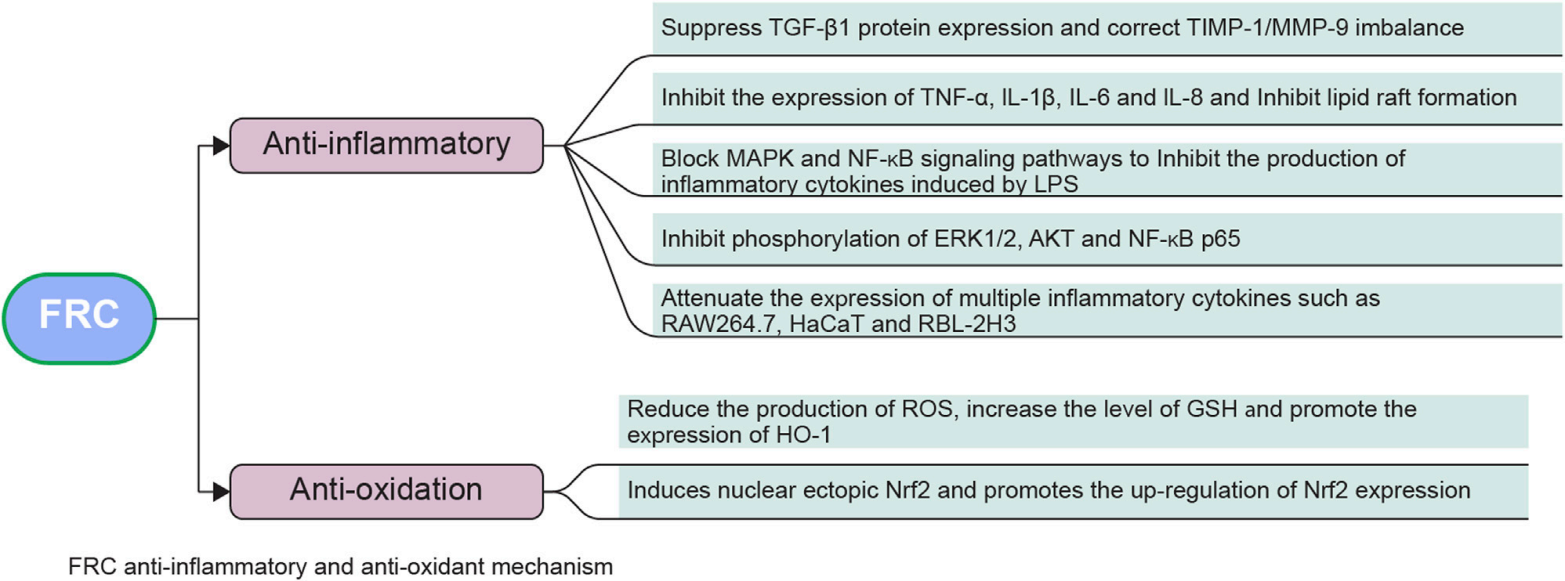
Anti-inflammatory and antioxidant mechanism of FRC. Abbreviations: TGF-β1, transforming growth factor β1; IL-1β, interleukin-1β; TIMP-1, matrix metalloproteinase inhibitor-1; MMP-9, matrix metalloproteinase-9; IL-6, interleukin-6; IL-8, interleukin-8; MAPK, mitogen-activated protein kinase; TNF-α, tumor necrosis factor-α; NF-κB, nuclear factor-activated B cells enhanced κ-light chain; LPS, lipopolysaccharide; ERK1/2, extracellular signal-regulated protein kinase 1/2; RAW264.7, monocyte/macrophage-like cell line; AKT, protein kinase B; NF-kB p65, nuclear factor/K gene binding nuclear factor antibody 65; HaCat, human immortalized keratinocyte cell line; ROS, reactive oxygen species; GSH, glutathione; HO-1, heme oxygenase-1; RBL-2H3, rat basophilic leukemia cell line; Nrf2, nuclear factor erythroid factor 2-related factor 2.

Part of the anti-inflammatory and antioxidant mechanisms of FRC are showed in Figure 4.

### 2.2 Pharmacological effects of FRT

Similar to FRC, FRT has a history of being used as medicine for over 2,000 years in China. Pharmacological studies have shown that FRT and its bulbs possess a broad spectrum of bioactivity, including antitussive, expectorant, antiulcer, anti-inflammatory, antioxidant, anticancer, neuroprotective, and analgesic activities (Nile et al., 2021).

#### 2.2.1 Cough suppression and expectoration

Two *in vivo* studies that investigated the cough suppressant effect of FRT showed a significant reduction in cough frequency (15 ± 7.6/5 min) and a prolonged period of remission (73.65 ± 43.02 t/s) after the oral administration of FRT-micronized powder in guinea pigs (Yan X. et al., 2012; Yan Z. et al., 2012). In addition, phenol red expectorant tests showed that FRT has a good expectorant effect and reduces sputum production significantly (Wang et al., 1993; Yan X. et al., 2012; Yan Z. et al., 2012; Qi et al., 2017).

#### 2.2.2 Anti-inflammatory effect

FRT has been applied in Chinese medicine since ancient times to treat inflammatory diseases such as bronchitis or pneumonia. Its ability to reduce inflammation is one of its most important pharmacological effects. Soengbeisine, ebeiedine, and verticine extracted from FRT can inhibit the expression and production of the mucin gene *MUC5AC* in the human respiratory epithelium, which is induced by epidermal growth factor (EGF), phorbol 12-myristate 13-acetate, or TNF-α (Kim et al., 2016). This is corroborated by the conventional use of FRT as a drug treatment for a broad range of inflammatory lung diseases. FRT-derived isoverticine (C6), puqiedine (C12), 2-monopalmitin (C13), zhebeiresinol (C14), and N-demethylpuqietinone were shown to reduce NF-κB expression in the human embryonic kidney cell line HEK293 and thus exert anti-inflammatory effects (Zhou et al., 2017). FRT extract significantly inhibited IL-6, IL-8, and TNF-α production in human mast cells (HMC-1) and attenuated the phosphorylation of three MAPK signaling channels (ERK, JNK, p38/MAPK) and NF-κB expression, thereby decreasing passive cutaneous anaphylaxis response in rats, indicating its inhibitory impact on anaphylaxis (Cho et al., 2011; Park et al., 2017). Peiminine was shown to inhibit both Nav1.7 and Kv1.3 ion channels, similar to lidocaine, suggesting its potential anti-inflammatory and analgesic effects (Xu et al., 2016).

**FIGURE 5:**
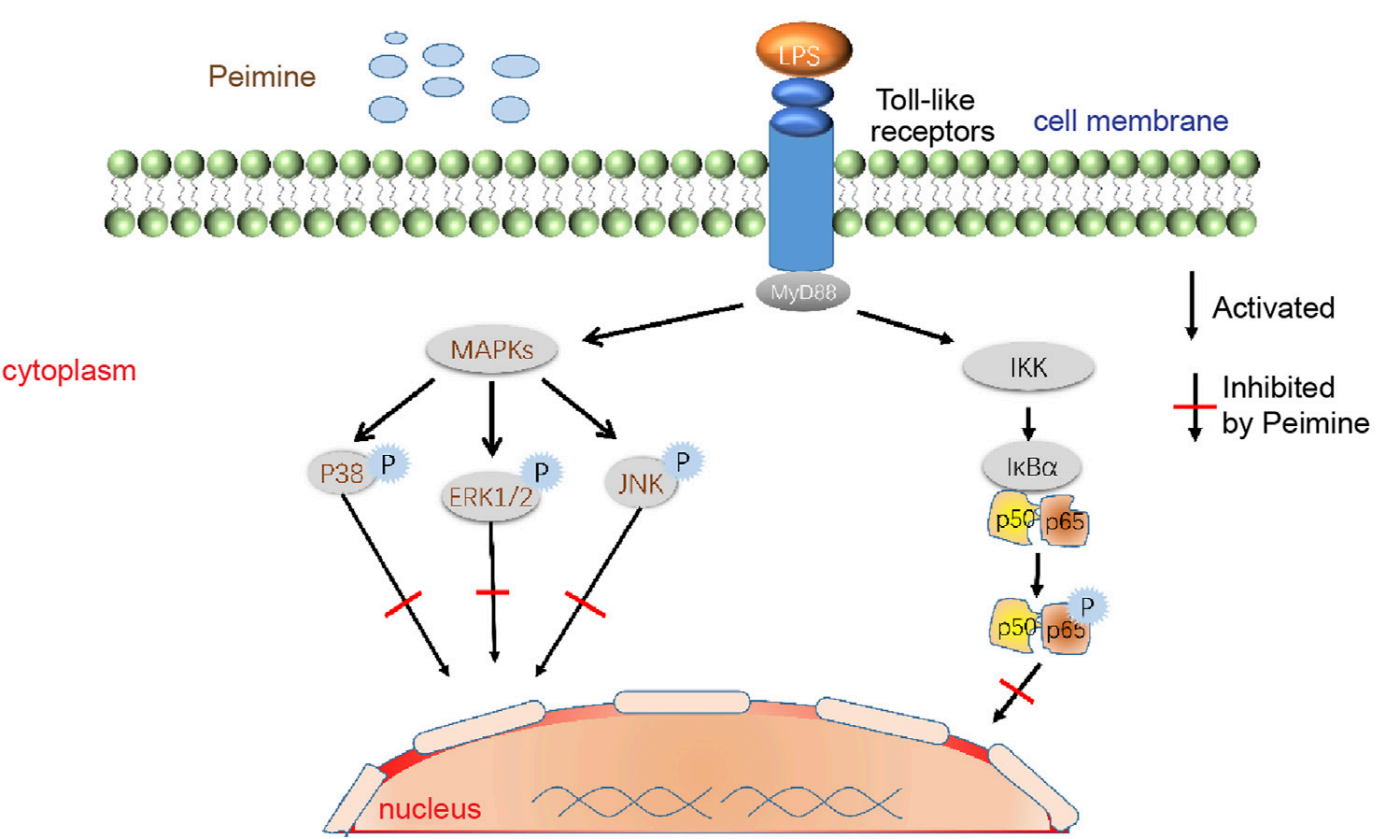
Modulation of MAPK and NF-κB signaling pathways by peimine. Abbreviations: LPS, lipopolysaccharide; IKK, inhibitor of nuclear factor kappa-B kinase; P, phosphorylation; MAPKs, mitogen-activated protein kinases; MyD88, myeloid differentiation factor 88; NF-κB, nuclear factor kappa-B; ERK, extracellular signal-regulated kinase; JNK, c-Jun N-terminal kinase. IκBα are significant members of the IκB (NF-κB inhibitor) clan, while NF-κB p50 and p65 are the most delegated dimer complexes in the nuclear transcription clan.

Part of the anti-inflammatory mechanism of peimine is shown in Figure 5.

#### 2.2.3 Antioxidant effect

By performing 2,2-diphenyl-1-picrylhydrazyl (DPPH) and 2,2′-azino-bis(3-ethylbenzothiazoline-6-sulfonic acid) (ABTS) free-radical scavenging tests and trivalent reduction activity assay, Ruan and others discovered that crude extracts and alkaloids derived from FRT exert significant antioxidant activity (Ruan et al., 2016). It was also found that polysaccharides extracted from FRT presented antioxidant effects at a concentration of 1 mg/ml (Ma, 2014). These results suggest that FRT has potential antioxidant activities; however, further research is needed to confirm this inference.

#### 2.2.4 Anticancer effect

Different studies have shown that FRT possesses antitumoral effects (Li et al., 2013; Liu et al., 2015). FRT was shown to reverse multidrug-resistance phenotypes in A549 lung adenocarcinoma (Li et al., 2013), HepG2 hepatocellular carcinoma (Liu et al., 2015), and MCF-7 breast cancer cells. The anticancer properties of FRT were also identified in two *in vivo* and *in vitro* experimental studies. FRT-derived peiminine inhibits cancer cell growth and movement and induces apoptosis by increasing the intracellular concentration of Ca^2+^ and phosphorylation induction of calcium/calmodulin-dependent protein kinase II (CaMKII) and JNK (Tan et al., 2020). Although FRT shows good promise as an antitumoral agent, more research on this topic is urgently needed.

**FIGURE 6:**
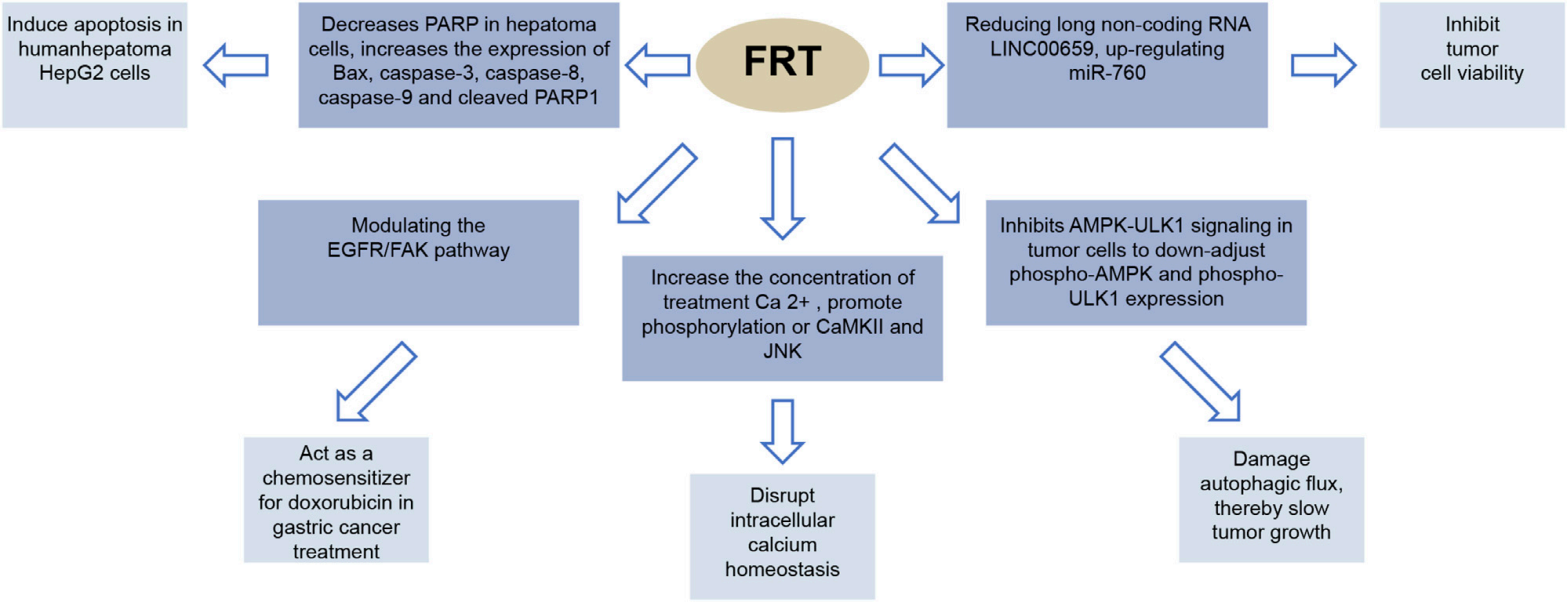
Anticancer effect of FRT. PARP (poly-ADP ribose polymerase) is a DNA repair leaven. It plays an important role in DNA damage repair and apoptosis. Bax is a pro-apoptotic protein member of the Bcl-2 family. EGFR is a member of the epidermal growth promoter receptor (HER) clan. FAK (focal adherent kinase) is a tyrosine kinase that is a local adhesion spot kinase. CaMKII is a calcium/calmodulin-dependent protein kinase II. JNK, also known as stress-activated protein kinase, plays an important role in a variety of physiological and pathological processes, including cell cycle, reproduction, apoptosis, and cellular stress.

Part of anti-cancer effect of FRT are showed in Figure 6.

#### 2.2.5 Other pharmacological effects

FRT-derived peiminine attenuates bleomycin-induced pulmonary fibrotic damage and infection by reducing the levels of circulating INF-γ and inhibiting MAPK/ERK signaling pathways (Guo et al., 2013). In a recent study, peiminine was found to reduce 6-hydroxydopamine-induced degeneration of dopaminergic nerve cells in the midbrain substantia nigra and enhance PTEN induced kinase 1 (PINK1)/parkin expression in order to alleviate apoptosis-related protein in the TGF-β signaling pathway (ARTS)-induced degradation of the X-linked apoptosis inhibitor, resulting in the inhibition of 6-hydroxydopamine-induced apoptosis and thus providing new insight that will be useful for the treatment of Parkinson’s disease (Hsu et al., 2021). Moreover, studies have shown that NF-κB and ERK1/2 signaling pathways are possible targets of peimine. Peimine was described to attenuate bone loss from an ovariectomized mouse by inhibiting the NFATc1, ERK, and NF-κB signaling pathways, thus suppressing the receptor activator of nuclear factor kappa-Β ligand (RANKL), which is involved in osteoclastogenesis (Zhu et al., 2021). *In vitro* experiments have shown that FRT-derived verticinone has a hypoglycemic effect mediated by increased insulin secretion, glucose uptake, and inhibition of carbohydrate hydrolase activity (Boojar et al., 2020). Another study has shown that fritillin A obtained from FRT inhibited the proliferation and stimulated apoptosis of KG-1a human acute myeloid leukemia cells (Zhang and Chen, 2015). Verticinone inhibits the activity of angiotensin converting enzyme, suggesting that *F. thunbergii* also has an antihypertensive impact (Oh et al., 2003).

#### 2.2.6 Common pharmacological effects

Because FRC and FRT have a high degree of similarity in terms of composition (Figure 2), they have comparable pharmacological effects, mainly in terms of anti-inflammatory and antitumoral properties.

The primary chemical constituents exhibiting anti-inflammatory effects are peimine and peiminine, which are common to both FRC and FRT. Peimine inhibits IL-1β-induced inflammation in mice chondrocytes by hindering the MAPK pathway (Chen et al., 2019). It also inhibits MAPK phosphorylation and NF-κB expression in mast cells, thus decreasing pro-inflammatory cytokine production (Park et al., 2017). Peimine further targets and inhibits nicotinic acetylcholine receptors with high affinity and may also be responsible for the anti-inflammatory properties of FR (Alberola-Die et al., 2021). Additionally, peiminine attenuates the expression of various inflammatory cytokines in RAW264.7, HaCaT, and RBL-2H3 cells (Lim et al., 2018). Peiminine inhibits the phosphorylation of NF-κB, AKT, ERK1/2, and p38 signaling pathways *in vivo* and *in vitro*, thus protecting against LPS-induced mammary-gland inflammation (Gong et al., 2018).

The main chemical constituents exhibiting antitumoral effects are also peiminine and peimine. Peiminine mediates cell cycle arrest by inhibiting Akt/GSk3β and AMPK-ULK1 signaling in glioblastoma multiforme, resulting in the suppression of autophagy, thereby inhibiting glioma growth (Zhao et al., 2018). Peiminine was further shown to reduce the expression of the long non-coding RNA LINC00659, which leads to upregulated miR-760 expression and ultimately inhibited colorectal cancer cell viability (Li et al., 2021). Peiminine also decreases poly (ADP-ribose) polymerase (PARP) activity in hepatoma cells, and increases the expression of Bax, caspase-3, caspase-8, caspase-9, and cleaved PARP1, ultimately leading to apoptosis (Chao et al., 2019). It can also act as a chemosensitizer for doxorubicin in gastric cancer treatment by modulating the EGFR/FAK pathway (Tang et al., 2018). Peimine was also shown to induce cancer cell apoptosis by disrupting intracellular calcium homeostasis *via* the Ca^2+^/CaMKII/JNK pathway (Tan et al., 2020).

## 3 Conclusion

In summary, we analyzed the places of origin, history of development, efficacy, chemical composition, and pharmacological effects of FRC and FRT, which are very similar in origin and composition and have very similar pharmacological effects. This study provides a basis for the clinical use of both herbal medicines. However, herbal medicines have multiple components and targets. As the pharmacological effects of many components have not been studied in depth, further research into the pharmacological mechanisms of each component is urgently needed. The components exhibit many pharmacological properties and thus show great potential in the development of new drugs. FRC and FRT are generally considered to be non-toxic herbal medicines that are free from side effects. Nevertheless, because few toxicological studies have been conducted on these two drugs, more research is needed to establish their clinical safety accurately.

## Data Availability

The original contributions presented in the study are included in the article/Supplementary Material, further inquiries can be directed to the corresponding author.
